# The role of lipid droplet formation in the protection of unsaturated fatty acids against palmitic acid induced lipotoxicity to rat insulin-producing cells

**DOI:** 10.1186/s12986-016-0076-z

**Published:** 2016-02-25

**Authors:** Thomas Plötz, Magnus Hartmann, Sigurd Lenzen, Matthias Elsner

**Affiliations:** Institute of Clinical Biochemistry, Hannover Medical School, 30623 Hannover, Germany

**Keywords:** Type 2 diabetes, Non-esterified fatty acids, Lipid droplets, Perilipin, Pancreatic β-cell, Peroxisomal β-oxidation

## Abstract

**Background:**

Type 2 diabetes is associated with increased plasma concentrations of non-esterified fatty acids (NEFAs), which trigger pancreatic β-cell dysfunction and apoptosis. Only long-chain saturated NEFAs induced lipotoxicity in rat insulin-producing cells in in vitro experiments, whereas unsaturated NEFAs were not toxic. Some unsaturated NEFAs even protected against lipotoxicity. In former studies it was suggested that long-chain unsaturated NEFAs, which induce the formation of lipid droplets, can cause sequestration of palmitic acid into lipid droplets. In the present structure-activity-relationship study the correlation between lipid droplet formation and the protection against palmitic acid induced lipotoxicity by unsaturated NEFAs in rat insulin-producing cells was examined.

**Methods:**

Rat insulin-producing RINm5F and INS-1E tissue culture cells were incubated in the presence of palmitic acid and unsaturated NEFAs with different chain lengths and different numbers of double bonds. The expression of the lipid droplet associated proteins perilipin 1 and 2 was repressed by the shRNA technique and the expression analyzed by qRT-PCR and Western blotting. Viability was measured by MTT assay and the accumulation of lipid droplets was quantified by fluorescence microscopy after Oil Red O staining.

**Results:**

Long-chain unsaturated NEFAs strongly induce the formation of lipid droplets in rat insulin-producing RINm5F and INS-1E cells. In RINm5F cells incubated with 11-eicosenoic acid (C20:1) 27 % of the cell area was covered by lipid droplets corresponding to a 25-fold increase in comparison with control cells. On the other hand the saturated NEFA palmitic acid only induced minor lipid droplet formation. Viability analyses revealed only a minor toxicity of unsaturated NEFAs, whereas the cells were markedly sensitive to palmitic acid. Long-chain unsaturated NEFAs antagonized palmitic acid induced lipotoxicity during co-incubation, whereby no correlation existed between protection and the ability of lipid droplet formation. Perilipin 1 and 2 expression was decreased after incubation with C20:1 to about 80 % by shRNA. For the protective effect of long-chain unsaturated NEFAs against lipotoxicity of saturated NEFAs repression of perilipin was not of crucial importance.

**Conclusions:**

Long-chain unsaturated fatty acids protected rat insulin-producing cells against lipotoxicity of saturated fatty acids. This protective effect was not dependent on lipid droplet formation. Thus lipid droplet formation is apparently not essential for the protective effect of unsaturated NEFAs against palmitic acid toxicity.

**Electronic supplementary material:**

The online version of this article (doi:10.1186/s12986-016-0076-z) contains supplementary material, which is available to authorized users.

## Background

Type 2 diabetes mellitus (T2DM) is one of the most prevalent metabolic diseases worldwide caused by insulin resistance of the peripheral tissues and impaired insulin secretion of pancreatic β-cells [1]. Physical inactivity and hypercaloric diets, rich in carbohydrates and saturated fats, are thought to be responsible for an increase in body weight and can cause the metabolic syndrome [2–4], which is characterized by obesity, hypertension and dyslipidemia, and often precedes the manifestation of T2DM. Chronically elevated plasma levels of non-esterified fatty acids (NEFAs), which are associated with the metabolic syndrome [5], can suppress insulin secretion and cause β-cell dysfunction and loss by apoptosis. This so-called lipotoxicity is subject to intensive research and scientific debate. Only long-chain saturated NEFAs induce lipotoxicity in rodent insulin-producing cells in in-vitro experiments, whereas unsaturated NEFAs are not toxic [6]. Short- and medium-chain fatty acids are metabolized in the mitochondrial β-oxidation, whereas long-chain fatty acids can be metabolized in the mitochondria but also in the peroxisomes when cells were exposed to high NEFA concentrations [7, 8]. Very long-chain fatty acids are exclusive substrates for the peroxisomal β-oxidation [8–10]. The electron transfer in the first step of the peroxisomal β-oxidation catalyzed by the enzyme acyl-CoA-oxidase results in high concentrations of hydrogen peroxide, which can cause cellular damage [8]. In contrast to other cell types pancreatic β-cells do not express the H_2_O_2_ inactivating enzyme catalase [11, 12]. This can explain why long-chain saturated fatty acids are harmful to rat insulin-producing β-cells and can cause apoptosis [7].

On the other hand unsaturated NEFAs are not toxic to rat insulin-producing cells and can even protect against lipotoxicity. The molecular mechanism of protection has not yet been fully elucidated, but experimental evidence has been provided that the formation of lipid droplets (LDs) by unsaturated NEFAs may be involved in the protection against lipotoxicity [13]. Specifically it has been suggested that long-chain unsaturated NEFAs can induce the formation of LDs, which may allow the sequestration of the toxic NEFA palmitic acid (PA) into these droplets. NEFAs converted into triglycerides and stored in LDs have been considered to be less toxic than NEFAs, which are readily metabolized [13]. For the formation of LDs perilipins are crucially important [14–17]. These proteins are associated with the LD surface and protect the stored triglycerides against hydrolysis by the hormone-sensitive lipases. Perilipins are thus important regulators of intracellular lipid storage [18, 19]. In the present study we therefore suppressed perilipin 1 and 2 expression in rat insulin-producing cell lines using the shRNA technique and analyzed the effects on LD formation and PA-induced toxicity. In a comprehensive approach we correlated the structural requirements of saturated and unsaturated NEFAs with different chain lengths between C12 and C24 with respect to LD formation and toxicity in rat insulin-producing cells.

Our results show that unsaturated NEFAs were able to antagonize palmitic acid induced lipotoxicity. However, we observed no correlation between protection and the ability of lipid droplet formation in rat insulin-producing cells.

## Methods

### Tissue culture of insulin-producing cells

The stable insulin-producing RINm5F cell line was established from a radiation-induced rat islet cell tumor by Gazdar et al. [20] and purchased from ATCC (American Type Culture Collection, Manassas, VA, USA; order# ATCC CRL-11605). Cells were cultured in RPMI 1640 medium supplemented with 10 mM glucose, 10 % (v/v) fetal calf serum (Biowest, Nuaillé, France), penicillin and streptomycin in a humidified atmosphere at 37 °C and 5 % CO_2_ [12]. The insulin-producing INS-1E cell line was clonally selected from a radiation-induced rat insulinoma and showed a stable phenotype for more than 100 passages [21, 22]. INS-1E cells were kindly provided by Prof. Claes Wollheim (University of Geneva, Switzerland). These cells were cultured in RPMI 1640 medium supplemented with 10 mM glucose, 10 % fetal calf serum (FCS), penicillin and streptomycin, 10 mM Hepes (Serva, Heidelberg, Germany), 2 mM glutamine, 1 mM sodium-pyruvate (Sigma-Aldrich, Munich, Germany), and 50 μM of 2-mercaptoethanol in a humidified atmosphere at 37 °C and 5 % CO_2_.

### NEFA incubation

For NEFA incubations a 50 mM stock solution was freshly prepared using 90 % ethanol as a solvent. NEFAs are bound to fatty acid free bovine serum albumin (BSA) in RPMI 1640 culture medium supplemented with 1 % fetal calf serum. The ratio between NEFA and BSA is 0.5 mM NEFA to 1 % BSA. For other NEFA concentrations the BSA concentration is adapted according to this ratio. C12:0 (dodecanoic acid/lauric acid), C12:1 (cis-5-dodecenoic acid), C14:0 (tetradecanoic acid/myristic acid), C14:1 (cis-9-tetradecenoic acid/myristoleic acid), C16:0 (hexadecanoic acid/palmitic acid), C16:1 (cis-9-hexadecenoic acid/palmitoleic acid), C18:0 (octadecanoic acid/stearic acid), C18:1 (cis-9-octadecenoic acid/oleic acid), C18:2 (cis-9, 12-octadienoic acid/linoleic acid), α-C18:3 (cis-9, 12, 15-octatrienoic acid/alpha-linolenic acid), γ-C18:3 (cis-6, 9, 12-octatrienoic acid/gamma-linolenic acid), C18:4 (cis-6, 9, 12, 15-octatetraenoic acid/stearidonic acid), C20:0 (eicosanoic acid/arachidic acid), C20:1 (cis-11-eicosenoic acid/gondoic acid), C20:2 (cis-11, 14-eicosadienoic acid), C20:3 (cis-8, 11, 14-eicosatrienoic acid), C20:4 (cis-5, 8, 11, 14-eicosatetraenoic acid/arachidonic acid), C20:5 (cis-5, 8, 11, 14, 17-eicosapentaenoic acid), C22:0 (docosanoic acid/behenic acid), C22:1 (cis-13-docosenoic acid/erucic acid), C22:2 (cis-13, 16-docosadienoic acid), C22:4 (cis-7, 10, 13, 16-docosatetraenoic acid), C22:6 (cis-4, 7, 10, 13, 16, 19-docosahexaenoic acid), C24:0 (tetracosanoic acid/lignoceric acid), C24:1 (cis-15-tetracosenoic acid/nervonic acid) were purchased from Sigma-Aldrich (Munich, Germany) or Larodan (Malmö, Sweden).

### Lipid droplet staining with Oil Red O

RINm5F cells were cultured for 24 h and afterwards exposed to 100 μM of different unsaturated NEFAs with different chain lengths and different numbers of double bonds (C18:1 to 18:4, C20:1 to C20:5, C22:1 to C22:6) for another 24 h. Cells were trypsinized and fixed in paraformaldehyde for 15 min at room temperature. Thereafter cells were stained with Oil Red O solution (Sigma-Aldrich, Munich, Germany) and washed twice with PBS (phosphate buffered saline). Lipid droplet formation was analyzed by fluorescence microscopy and the area within the cells was quantified by the use of the Olympus xcellence software (Olympus, Hamburg, Germany) at 546 nm excitation and 580 nm emission. For each incubation condition five to seven coincidentally selected images, each containing 30 to 100 cells, were used to quantify the proportion of the lipid droplet area to the total cell area with the phase analysis module of the xcellence software.

### Assessment of cell viability

RINm5F insulin-producing cells were seeded at 2.5 × 10^4^ cells/well in 100 μl culture medium onto 96-well plates and allowed to attach for 24 h before they were incubated at 37 °C with combinations of different unsaturated NEFAs and PA (palmitic acid/hexadecanoic acid/C16:0) for further 24 h. Cell viability and the ability to protect against PA-induced toxicity was determined by a microplate-based MTT assay (3-(4,5-dimethylthiazol-2-yl)-2,5-diphenyl tetrazolium bromide, Serva, Heidelberg, Germany) [23].

### Lentivirus preparation and transduction of insulin-producing cells

Lentiviral vector particles were produced as described [24]. In brief, 5x10^6^ 293FT cells were transfected with the packaging plasmid pPAX2 (37.5 μg), the envelope plasmid pcDNA-MDG (7.5 μg), and the transfer plasmids pLV-U6-sh-rPlin1, pLV-U6-sh-rPlin2 or pLV-U6-sh-NTC (22.5 μg) by calcium phosphate precipitation. The transfer plasmids were produced by Sirion Biotech (Munich, Germany) and validated for suppression efficiencies > 85 %. The virus particles were harvested from the culture medium 48 h after transfection and purified by ultrafiltration columns at 3000 g for 25 min (Amicon Ultra Ultracel-100 K, Millipore, Schwalbach, Germany). Virus titers (typically 5x10^7^ infectious particles) were quantified by a TaqMan qPCR assay as described elsewhere [24]. RINm5F-Tet3G and INS-1E-Tet3G cells were transduced with either the sh-rPlin1, sh-rPlin2 or sh-NTC virus with a multiplicity of infection (MOI) followed by a selection with puromycin (2.5 μg/ml).

### Gene expression analyses

RNA from RINm5F and INS-1E cells was isolated with the RNeasy Kit (Qiagen, Hilden, Germany) according to the manufacturer’s instructions. The quality of the total RNA was verified by comparing the intensities of the 28S and 18S rRNA bands in agarose gel electrophoresis. Two microgram total RNA was reverse transcribed into cDNA using the RevertAid H Minus M-MuLV Reverse Transcriptase (Thermo Fisher Scientific, Schwerte, Germany) and random hexamer primers (Life Technologies, Karlsruhe, Germany). The *Plin1* or *Plin2* expression in 10 ng cDNA was quantified by a SYBR Green based assay (GoTaq Green Master Mix; Promega, Mannheim, Germany) and performed on an Opticon fluorescence detection system (Biorad, Munich, Germany) with the following protocol: Samples were initially denaturated at 95 °C for 2 min followed by up to 40 PCR cycles. Each PCR cycle comprised a denaturation at 94 °C for 30 s, an annealing at 60 °C for 30 s, and an extension at 72 °C for 30 s. The specificity of the amplification was verified by melting point analysis. For each sample amplification was performed in triplicate. The *Plin1* and *Plin2* expression data were normalized against the geometric mean of the three reference genes *actin beta* (*Actb*), peptidylprolyl isomerase A (*PPIA*), and *TATA box binding protein* (*TBP*) with the software qBasePlus (Biogazelle, Zwijnaarde, Belgium). Primers for qPCR analyses were synthesized by Life Technologies (Karlsruhe, Germany): Plin1-fw (5′ GATTCTGCTTTGCAGCGTGAA 3′), Plin1-rv (5′ CAGGACTCTCTGGAGCACATTC 3′), Plin2-fw (5′ TCGTCTCTCAGCTCTCCTGT 3′), Plin2-rv (5′ TAGGTGGAGCTCACCAAGGG 3′), Actb-fw (5′ GCGTCCACCCGCGAGTACAA 3′), Actb-fw (5′ TTGCACATGCCGGAGCCGTT 3′), PPIA-fw (5′ TTGCAGACGCCGCTGTCTCTT 3′), PPIA-rv (5′ TGGAACTTTGTCTGCAAACAGCTCG 3′) TBP-fw (5′ CAGCTCGGCGCACCGTACAT 3′), TBP-rv (5′ TCTGGGTTATCGTCACGCACCA 3′).

### Western blot analyses

Whole cell extracts were prepared in radioimmune precipitation assay buffer according to the manufacturer’s recommendation (Sigma) supplemented with complete protease inhibitor mixture (Roche Diagnostics, Mannheim, Germany). Protein content was determined by the BCA assay (Thermo Fisher Scientific, Rockford, IL, USA). 30 μg of total protein were separated by a 10 % SDS-PAGE and electroblotted to nitrocellulose membranes (GE Healthcare, Buckinghamshire, UK). Nonspecific binding sites of the membranes were blocked with 5 % nonfat dry milk for 1 h at room temperature. The membranes were incubated with specific primary antibodies overnight at 4 °C. The following antibodies from Santa Cruz Biotechnology (Santa Cruz, CA, USA) were used: anti-Perilipin 1 (H-300, diluted 1:2000), anti-Perilipin 2 (H-80, diluted 1:200), and anti-β-actin (sc-1615, diluted 1:250). The excess of primary antibody was removed by three washing steps with washing buffer (PBS, 0.1 % Tween 20, 0.1 % BSA). Subsequently, the membranes were incubated with peroxidase-labeled secondary anti-rabbit antibodies at a dilution of 1:20,000 at room temperature for 1 h. Protein bands were visualized by chemiluminescence using the ECL detection system (GE Healthcare). As a loading control the expression of β-actin was analyzed after stripping the blots with Re-blot Pus solution (Merck-Millipore, Darmstadt, Germany) according to the manufacturer’s manual. Protein band intensity was quantified and normalized to β-actin though densitometry with the Gel-Pro Analyzer program (version 6.0, Media Cybernetics, Silver Spring, MD, USA).

### Statistical analysis

Data are expressed as means ± SEM. Statistical analyses were performed using ANOVA plus Dunnett’s or Bonferroni’s test for multiple comparisons, unless stated otherwise (GraphPad Prism5, San Diego, CA, USA).

## Results

### Induction of lipid droplet formation by NEFAs in insulin-producing cells

**Fig. 1:**
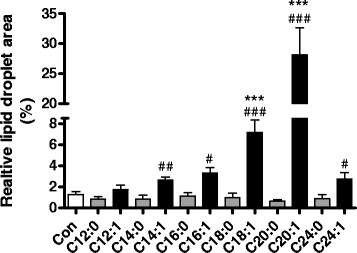
Lipid droplet formation in rat insulin-producing RINm5F cells after incubation with saturated and monounsaturated NEFAs of increasing chain length. RINm5F cells were incubated with different medium- and long-chain saturated and monounsaturated NEFAs (100 μM) for 24 h. Thereafter cells were fixed and stained with Oil Red O. Lipid droplet formation was analyzed by fluorescence microscopy and quantified using the software xcellence rt. Data are means ± SEM of *n* = 3. ***p < 0.001 compared to control cells (Dunnett’s Multiple Comparison Test). #*p* < 0.05, ##*p* < 0.01, ###*p* < 0.005 compared to saturated NEFA with the same chain length (Student’s *t*-test)

Long-chain monounsaturated NEFAs (C18:1, C20:1, C22:1) strongly induced the formation of LDs in rat insulin-producing RINm5F cells. More than 25 % of the cell area of RINm5F cells incubated with the long-chain NEFA gondoic acid (C20:1, 100 μM) was covered by LDs, which corresponds to a 16-fold increase in comparison to untreated cells. On the other hand incubation with medium-chain and long-chain monounsaturated NEFAs (C12:1, C14:1, C16:1) and with a very long-chain NEFA (C24:1) showed no significant increase in LD formation in comparison to control cells (Fig. 1). In contrast to unsaturated NEFAs none of the analyzed saturated NEFAs (C12:0 to C24:0) caused significant increases in LD content in RINm5F cells.

### Effect of saturation level of unsaturated NEFAs on lipid droplet formation in insulin-producing cells

**Fig. 2:**
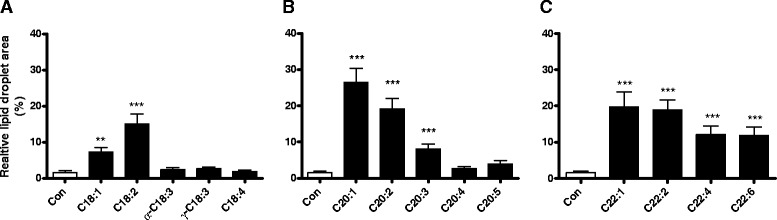
Lipid droplet formation in rat insulin-producing RINm5F cells after incubation with unsaturated NEFAs with a different degree of unsaturation. RINm5F cells were incubated with C18:x (**a**), C20:x (**b**) and C22:x (**c**) NEFAs (100 μM) with different numbers of double bonds for 24 h. Thereafter cells were fixed and stained with Oil Red O. Lipid droplet formation was analyzed by fluorescence microscopy and quantified using the software xcellence rt. Data are given as means ± SEM from 5 to 8 individual experiments. **p < 0.01, ***p < 0.001 compared with control cells (Dunnett’s Multiple Comparison Test)

In the group of unsaturated NEFAs with the chain length of C18 with different numbers of double bonds (C18:x) only C18:1 and C18:2 revealed a significant up to 15 % increase in the cell area covered with LDs (Fig. 2a). However, the unsaturated NEFAs C18:3 and C18:4 showed no increase in LD formation compared to the control condition. With 27 % LDs of the cell area C20:1 induced maximal lipid droplet formation. With an increasing number of double bonds the LD area declined to < 4 % after incubation with C20:4 or C20:5 (Fig. 2b), which was not significantly higher than in the control condition. The unsaturated C22:x NEFA group revealed the same tendency with the greatest LD formation after incubation with the monounsaturated C22:1 and a steady decrease with increasing numbers of double bonds up to C22:6 (Fig. 2c).

### Antagonistic effect of saturated fatty acids against lipid droplet formation

**Fig. 3:**
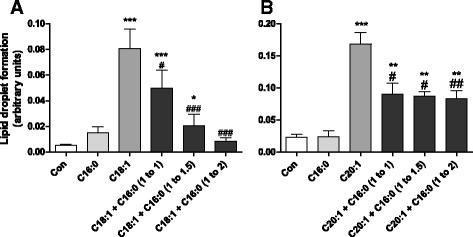
Lipid droplet formation in co-incubation experiments with unsaturated NEFAs in combination with increasing PA concentrations. RINm5F cells were incubated with 100 μM of the unsaturated NEFAs C18:1 (**a**) or C20:1 (**b**) in combination with increasing concentrations of the saturated NEFA C16:0 for 24 h. Thereafter cells were fixed, stained with Oil Red O and analyzed by fluorescence microscopy. Lipid droplet formation per cell was quantified by the software xcellence rt. *p < 0.05, **p < 0.01, ***p < 0.001 compared to control cells (Dunnett’s Multiple Comparison Test). ^#^ *p* < 0.05, ^##^ *p* < 0.01, ^###^ *p* < 0.005 compared to unsaturated NEFA only (Student’s *t*-test)

Long-chain unsaturated NEFAs induced LD formation, whereas long-chain saturated NEFAs were not able to form LDs. A co-incubation experiment of both saturated and unsaturated NEFAs revealed a decline of LD formation typically induced by long-chain unsaturated NEFAs. Palmitic acid (C16:0), which is the physiologically and nutritionally most important saturated fatty acid, antagonized the LD formation by C18:1 and to a lesser extent by C20:1 (Fig. 3a-b). The antagonistic effect increased with the increase of the proportion of C16:0 in the mixtures with C18:1 and C20:1.

### Toxicity of long-chain NEFAs in insulin-producing cells

Long-chain saturated NEFAs at a 100 μM concentration were highly toxic to insulin-producing RINm5F cells. On the other hand the corresponding unsaturated NEFAs with the same chain lengths showed only a minimal or no significant toxicity at a concentration of 100 μM compared to the toxic effect of palmitic acid with a viability of 31 % (Additional file 1: Figure S1). Generally no correlation between the toxicity and the number of double bonds could be identified.

### Protective capacity of long-chain unsaturated NEFAs against the toxicity of palmitic acid in insulin-producing cells in dependence on chain length and saturation level

The protective capacity of long-chain unsaturated NEFAs was analyzed in co-incubation experiments of 200 μM PA with increasing concentrations of unsaturated NEFAs to determine the unsaturated NEFA concentration necessary to achieve a 50 % viability reduction (Table 1). The best protection against PA-induced toxicity in RINm5F insulin-producing cells was provided by C20:5 with a half-maximally effective protective concentration of 5.4 μM. Generally NEFAs of the C20:x group revealed the highest protective potency. In the NEFA groups of C20:x and C22:x the monounsaturated NEFAs were less protective than the polyunsaturated NEFAs. A concentration of 17.2 μM for C20:1 and of 88.6 μM for C22:1 respectively was required to antagonize PA toxicity by 50 %. With a rise in the number of double bonds in these two groups the protective potential increased. The group of C18:x unsaturated NEFAs revealed the weakest protection of all tested groups. There were no significant differences in the protective potency against PA-induced toxicity between the omega-9 fatty acid oleic acid (C18:1), the omega-6 fatty acid γ-linolenic acid (γ-C18:3) and the omega-3 fatty acid α-linolenic acid (α-C18:3). The lowest protective potency exhibited the omega-3 fatty acid C18:4.

**Table 1:** Protective effect of unsaturated NEFAs against PA-induced toxicity and relative lipid droplet area linked with the correlation coefficient for each group

NEFA	Half-maximal protective concentration (μM)	Lipid droplet area after incubation with 100 μM NEFA (% of cell area)	Correlation coefficient for each group	*P* value for each group
C18:1	32.8 ± 2.2 (6)	7.3 ± 1.3 (5)	C18-FFA: +0.52	C18-FFA: 0.37
C18:2	11.4 ± 1.2 (5)	15.1 ± 2.8 (5)		
α-C18:3	24.0 ± 1.8 (4)	2.4 ± 0.6 (5)		
γ-C18:3	27.2 ± 1.6 (4)	2.6 ± 0.5 (5)		
C18:4	> 100.0 (5)	1.9 ± 0.3 (5)		
C20:1	17.2 ± 1.6 (6)	26.5 ± 4.9 (5)	C20-FFA: −0.63	C20-FFA: 0.25
C20:2	6.0 ± 0.6 (4)	19.2 ± 2.9 (5)		
C20:3	10.4 ± 1.2 (4)	8.1 ± 1.3 (5)		
C20:4	8.6 ± 0.6 (4)	2.7 ± 0.6 (5)		
C20:5	5.4 ± 0.6 (4)	3.9 ± 1.0 (5)		
C22:1	88.6 ± 22.8 (6)	19.7 ± 4.1 (5)	C22-FFA: −0.89	C22-FFA: 0.11
C22:2	42.0 ± 6.8 (4)	18.9 ± 2.7 (5)		
C22:4	8.6 ± 1.4 (5)	12.1 ± 2.5 (5)		
C22:6	10.8 ± 3.2 (5)	11.8 ± 2.4 (5)		
Total value			0.04	0.89

The protective concentration of the unsaturated NEFAs was determined in co-incubation experiments for 24 h with 200 μM PA resulting in a 50 % cell viability measured by MTT assay. The protective effects were calculated by nonlinear regression analyses. Lipid droplet and MTT data are means ± SEM with the number of independent experiments in parenthesis. Correlation coefficients and *P* values were determined by Pearson correlation

### Lack of correlation between protective potency against PA-induced toxicity and lipid droplet formation in insulin-producing cells

The most efficient unsaturated NEFA with respect to the ability to form lipid droplets was C20:1, which mediated only a moderate protection against PA-induced toxicity. The highest protective potency against PA-induced toxicity revealed C20:5, but it showed no significant increase in lipid droplet formation compared to control conditions without NEFAs. To examine, whether there was a correlation between the protective potency and lipid droplet formation in the different unsaturated NEFA groups (C18:x, C20:x, C22:x) the corresponding correlation coefficients and the *p* values were calculated. In none of the groups the correlation coefficient was significant (Table 1). A calculation of an overall coefficient between lipid droplet formation and the protective potency of the different NEFA groups revealed a correlation coefficient of 0.04.

### Gene expression analyses of perilipin 1 or 2 in insulin-producing cells after suppression of perilipin 1 or 2

**Fig. 4:**
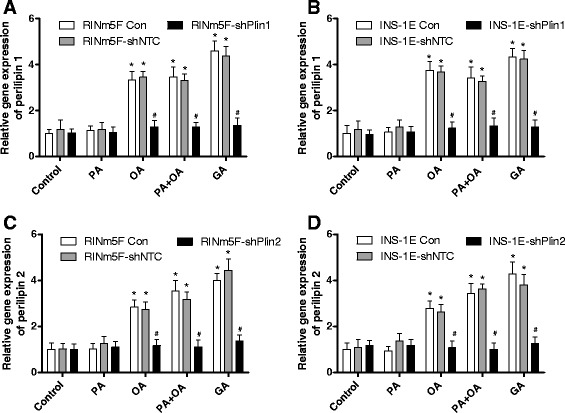
Gene expression analysis of perilipin 1 or 2 suppressed RINm5F and INS-1E cells. Perilipin 1 and 2 expression in RINm5F (**a**, **c**) and INS-1E (**b**, **d**) cells was stably suppressed by the shRNA technique after lentiviral transduction. Cells were incubated with 100 μM palmitic acid (PA), 100 μM oleic acid (OA), a combination of both, or 100 μM gondoic acid (GA) or without NEFAs (Con) for 24 h. Thereafter RNA was isolated and reverse transcribed into cDNA. Gene expression of perilipin 1 and perilipin 2 was measured by qRT-PCR and normalized to the reference genes *PPIA*, *α-tubulin* and *β-actin* using qBase + software (Biogazelle). Data are means ± SEM of *n* = 4. ^*^ *p* < 0.01 compared to control cells without NEFAs (ANOVA/Tukey Multiple Comparison Test). ^#^ *p* < 0.01 compared with control cells (ANOVA/Tukey Multiple Comparison Test)

To verify the efficiency of the shRNA mediated knockdown of perilipin 1 or 2 (shRNA-Plin1 or shRNA-Plin2) gene expression in insulin-producing RINm5F and INS-1E cells was analyzed after incubation with PA, OA (oleic acid/cis-9-octadecenoic acid/C18:1), a combination of PA and OA, or GA (gondoic acid/cis-11-eicosenoic acid/C20:1). Control INS-1E and RINm5F cells as well as non-target shRNA control cells showed a significant 3- to 4-fold increase in perilipin 1 gene expression after incubation with OA, PA + OA, or GA in comparison to control conditions without NEFAs. In shRNA-Plin1 cells no significant increase was detectable (Fig. 4a-c). Similar results were obtained in shRNA-Plin2 cells. Only in control cells and non-target shRNA control cells a significant increase in perilipin 2 expression was detectable, whereas the gene expression in shRNA-Plin2 was not significantly increased after incubation with OA, PA + OA, or GA in comparison to control conditions (Fig. 4b-d).

**Fig. 5:**
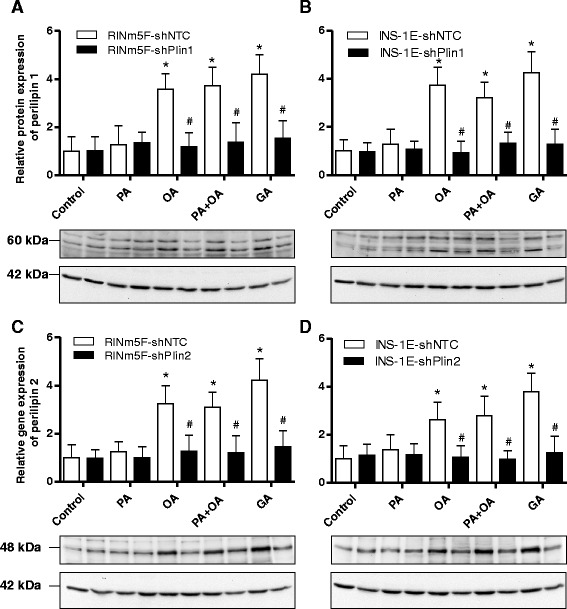
Western blot analyses of perilipin 1 or 2 suppressed RINm5F and INS-1E cells. Perilipin 1 and 2 expression in RINm5F (**a**, **c**) and INS-1E (**b**, **d**) cells was stably suppressed by the shRNA technique after lentiviral transduction. Cells were incubated with 100 μM palmitic acid (PA), 100 μM oleic acid (OA), a combination of both, or 100 μM gondoic acid (GA) or without NEFAs (Con) for 24 h. Thereafter, 30 μg protein of total whole cell extracts were separated by a SDS-PAGE and electroblotted on nitrocellulose membranes. Specific protein detection was carried out with a primary anti-perilipin 1 or anti-perilipin 2 antibody and a horseradish peroxidase labelled anti-rabbit IGG secondary antibody. After visualization of the protein bands by chemiluminescence the perilipin 1 and 2 bands were quantified and normalized to β-actin bands. Shown are means of ± SEM of *n* = 3 independent immunoblots and one representative blot for each cell line. ^*^ *p* < 0.01 compared to control cells without NEFAs (ANOVA/Tukey Multiple Comparison Test). ^#^ *p* < 0.01 compared with control cells (ANOVA/Tukey Multiple Comparison Test)

In addition to the gene expression quantification the mRNA level, perilipin 1 and 2 protein expression in insulin-producing RINm5F and INS-1E cells was analyzed by immunoblotting (Fig. 5). In Western blot analyses two bands in the range between approx. 60 kDa and 65 kDa were detectable for perilipin 1. This finding confirms earlier immunoblotting analyses of INS-1E cells [13]. For perilipin 2 the immunoblotting revealed a specific band at a molecular weight of 48 kDa. Through shRNA mediated suppression of perilipin 1 or perilipin 2 a 60 to 70 % reduction both on the mRNA and protein level was achieved after incubation with OA, PA + OA, or GA, when compared to non-targeted control cells (Fig. 5). PA alone did not induce expression of perilipin 1 or 2. This low expression level was also not affected by shRNA mediated perilipin suppression.

### Quantification of lipid droplet formation in insulin-producing cells after exposure to oleic acid

**Fig. 6:**
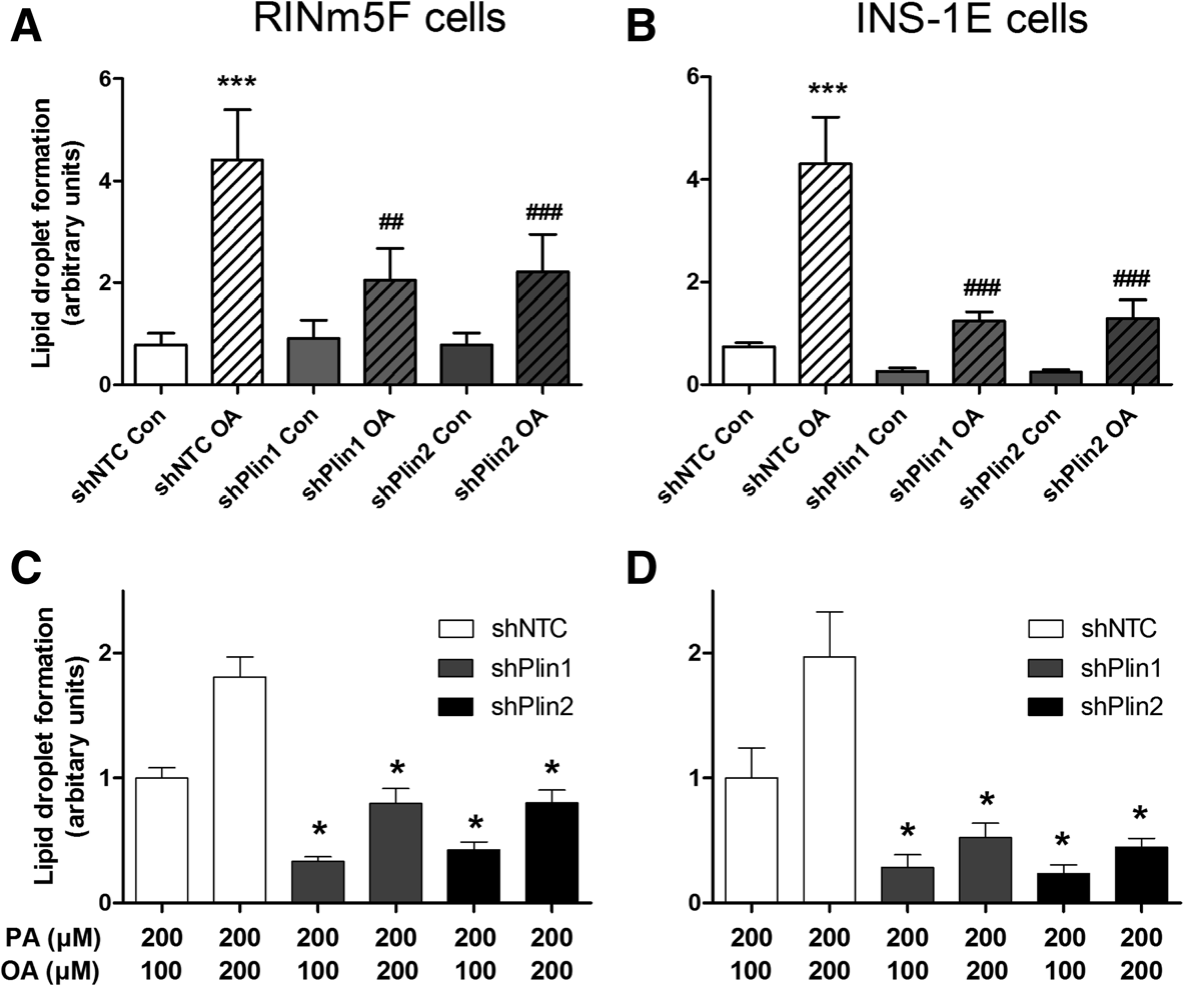
Effect of perilipin 1 or 2 suppression in insulin-producing RINm5F and INS-1E cells on lipid droplet formation after OA and PA incubation. For the suppression of perilipin 1 and 2 RINm5F (**a**, **c**) and INS-1E (**b**, **d**) cells were transduced lentivirally with the respective shRNA constructs or as a control with a non-target shRNA (shNTC). Cells were incubated with the indicated NEFAs for 24 h and after fixation stained with Oil Red O. Lipid droplet formation was analyzed by fluorescence microscopy and quantified using the software xcellence rt. **a**, **b** Cells were incubated with 100 μM oleic acid (C18:1, OA) or under control conditions (Con). Data are presented as means ± SEM of 4 to 5 independent experiments. ^##^ *p* < 0.01; ^###^ *p* < 0.001 compared with shNTC cells after incubation with OA (ANOVA/Tukey Multiple Comparison Test). ***p < 0.001 compared with non-OA-incubated controls (ANOVA/Tukey Multiple Comparison Test). **c**, **d** Cells were incubated with 200 μM palmitic acid (PA) in combination with 100 or 200 μM OA. Data are presented as means ± SEM of 4 independent experiments. *p < 0.05; compared with shNTC cells with the respective PA and OA concentrations (ANOVA/Tukey Multiple Comparison Test)

In order to verify the effect of perilipins on LD formation shRNA-Plin1 or shRNA-Plin2 RINm5F and shRNA-Plin1 or shRNA-Plin2 INS-1E cells were incubated with the physiologically most important NEFAs, the saturated palmitic acid, (PA, C16:0) and the monounsaturated oleic acid (OA, C18:1) as well as a combination of both. Maximal LD formation was induced in RINm5F and INS-1E control cells or shRNA non-target controls (shNTC) after OA incubation in comparison to cells incubated without this NEFA (Fig. 6a and b). In shRNA-Plin1 or shRNA-Plin2 cells C18:1 exposure resulted in a non-significant increase in LD formation in comparison to cells not incubated with OA. Furthermore shRNA-Plin1 or shRNA-Plin2 cells generated significantly fewer LDs in response to OA when compared to non-suppressed perilipin cells. The reduction in LD formation was in INS-1E-shPlin cells approx. 80 % (Fig. 6a) and in RINm5F-shPlin cells approx. 50 % (Fig. 6b). Incubation of RINm5F and INS-1E cells with knockdown of perilipin 1 or 2 in the presence of PA (200 μM) plus OA (100 or 200 μM) also caused a significant reduction of lipid droplet formation in the range of 50–70 % in comparison with the respective shNTC control cells (Fig. 6c and d). PA (200 μm) alone did not cause lipid droplet formation (data not shown).

### Palmitic acid-induced lipotoxicity in perilipin suppressed insulin-producing cells

**Fig. 7:**
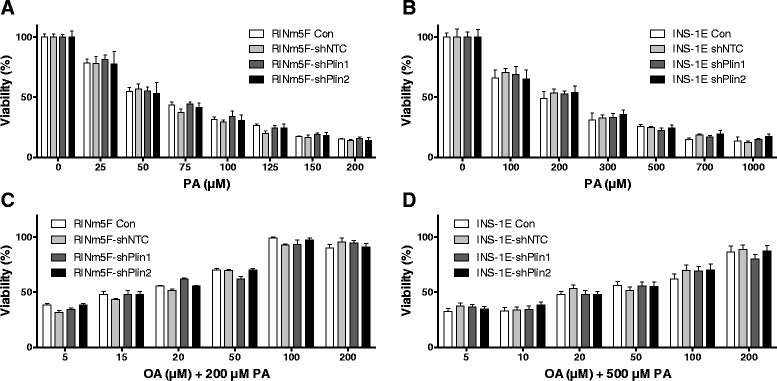
PA-induced lipotoxicity and OA protection against PA-induced lipotoxicity in perilipin 1 or 2 suppressed insulin-producing RINm5F and INS-1E cells. Perilipin 1 or 2 suppressed RINm5F cells were incubated with increasing concentrations of the saturated NEFA C16:0 (PA) (**a**) or co-incubated with 200 μM PA and increasing concentrations of the monounsaturated NEFA C18:1 (OA) (**c**). Perilipin 1 or 2 suppressed INS-1E cells were incubated with increasing concentrations of the saturated NEFA C16:0 (PA) (**b**) or co-incubated with 500 μM PA and increasing concentrations of the monounsaturated NEFA C18:1 (OA) (**d**). The cell viability was measured by MTT assay after 24 h. Data are means ± SEM of *n* = 4 to 6. Significance was tested against control cells (ANOVA/Tukey Multiple Comparison Test)

Perilipin suppressed RINm5F and INS-1E cells were used to analyze differences in the sensitivity against PA-induced toxicity. Neither perilipin 1 suppression nor perilipin 2 suppression showed a significantly higher sensitivity when compared to non-target shRNA control cells after incubation with the toxic saturated NEFA PA (C16:0) (Fig. 7a and b). Furthermore the cell lines were co-incubated with 200 μM PA and increasing concentrations of OA (up to 200 μM) demonstrating the protective effect of the monounsaturated NEFA OA on PA-induced toxicity. In these experiments there were also no significant differences in toxicity detectable between perilipin 1 or 2 suppressed cell lines detectable (Fig. 7c and d). Incubation with OA (up to 500 μM) alone did not induce significant toxicity in the cell lines (data not shown).

## Discussion

Obesity and the metabolic syndrome are associated with increased plasma concentrations of non-esterified fatty acids (NEFAs) concentrations in the circulation, which can cause T2DM by inducing insulin resistance in peripheral tissues as well as damage to pancreatic β-cells [1, 2]. In several in vitro studies on lipotoxicity in rodent β-cells long-chain saturated NEFAs were found to be responsible for the induction of apoptosis by ER-stress [25–27] or the formation of toxic reactive oxygen species (ROS) [7, 28–30]. In contrast, long-chain unsaturated NEFAs are less toxic or non-toxic to rat β-cells and can even provide protection against the toxicity induced by long-chain saturated NEFAs. We recently reported that the PA-induced formation of cytotoxic H_2_O_2_ in the peroxisomal β-oxidation is inhibited by long-chain unsaturated NEFAs [27]. Pancreatic β-cells do not express the H_2_O_2_ detoxifying enzyme catalase which contributes to the exceptional sensitivity towards ROS mediated toxicity of this cell type [11, 31].

Another concept offered to explain the protection of long-chain unsaturated NEFAs against lipotoxicity is the formation of cytosolic lipid droplets (LDs). NEFAs in the cytosol can be sequestrated to LDs after esterification in triglycerides and could therefore not express their cytotoxic action [13, 32, 33]. PA is known to be incorporated into LDs to a significantly lesser extent and evokes a greater lipotoxic effect than OA [32–34]. Furthermore, when cells are incubated with PA and OA simultaneously, OA induces LD formation and facilitates the incorporation of PA into the LDs, and thereby is thought to decrease the lipotoxic action of PA [33]. It was therefore the aim of the present study to verify if this hypothesis is applicable not only to OA but also to other unsaturated NEFAs with different chain lengths and saturation levels in rat insulin-producing cells.

In a comprehensive experimental approach we analyzed for the first time a group of 16 different saturated as well as mono- and polyunsaturated NEFAs with chain lengths ranging from C12 to C24 by fluorescence microscopy for their ability to form LDs in insulin-producing cells. Only long-chain mono- and polyunsaturated NEFAs were found to cause LD formation whereas the saturated NEFAs were ineffective. Such a difference had been reported in previous studies only between the unsaturated NEFA OA and the unsaturated NEFA PA [13, 32, 33, 35]. Our detailed comparison of different mono- and polyunsaturated NEFAs with chain lengths of C18, C20 and C22 revealed no correlation (correlation coefficient *r* = 0.04) between extent of NEFAs saturation and LD formation.

These results are in disagreement with the previously proposed hypothesis that LD formation is responsible for the protective effect of unsaturated NEFAs against toxicity of long-chain saturated NEFAs [13, 32, 33, 35]. To obtain further evidence for a lack of association between LD formation and a possible protective effect of unsaturated NEFAs against the toxicity of the saturated NEFA PA we determined in RINm5F insulin-producing cells in co-incubation experiments with 200 μM PA the half maximal protective concentration for each of the 14 unsaturated NEFAs with different chain lengths and saturation levels and correlated these data with the LD formation. Interestingly, there was no correlation detectable between toxicity and LD formation. This conclusion is in contradiction to the hypothesis of a crucial role of LDs for the protection against PA toxicity [13, 31, 32], which was based only on analyses of the effects of PA, OA and linoleic acid [13, 35]. Thus a valid conclusion must be based upon the analyses of more than these three NEFAs.

Additional evidence that LD formation is not a crucial protective mechanism against lipotoxicity was provided by the perilipin 1 and 2 repression experiments in insulin-producing cells. Perilipin repression was used as an additional experimental tool to study the effect of LD formation on lipotoxicity. Perilipins are proteins which are involved in the biogenesis of LDs and located in the phospholipid mono layer of the LDs [16, 17]. Like other investigators [13] we were able to detect perilipin 1 and 2 gene expression in rat insulin-producing RINm5F and INS-1E cells on the mRNA as well as on the protein level. Incubation with OA, PA + OA, or GA led to an approx. 3 to 4-fold increase in perilipin 1 and 2 mRNA and protein expression. This induced perilipin 1 and 2 expression was diminished by the shRNA technique by 60 % to 80 %. This gene suppression caused also a 50-80 % reduction in LD formation in insulin-producing cells indicating that perilipins are involved in LD formation. If LD formation would be of crucial importance for lipotoxicity of long-chain saturated NEFAs it should be expected, that the protection of OA against PA-induced toxicity was reduced in perilipin 1 and 2 repressed cells which was not the case in our experiments. On the other hand we obtained also no experimental evidence for a short-term detrimental effect on cell viability of LD storing unsaturated fatty acids. This does not exclude that long-term intracellular storage of fatty acids in the form of triglycerides may have unfavorable effects on the cellular function; in particular in diseases affecting other organs such as non-alcoholic fatty liver [36] and cardiovascular diseases [37], which go along with pathological intracellular fat accumulation.

## Conclusions

The results document convincingly that LD formation plays no essential role in the protective effect of unsaturated NEFAs against the toxicity of palmitic acid in rat insulin-producing cells.

## Additional file

Additional file 1: Figure S1. Viability of long-chain unsaturated NEFAs in comparison to the long-chain saturated NEFA PA (C16:0). RINm5F cells were incubated with 100 μM of the long-chain saturated NEFA or 100 μM of the unsaturated NEFAs as depicted for 24 h. Thereafter viability was measured by MTT assay. Data are means ± SEM of *n* = 5 to 8. **p* < 0.05, ***p* < 0.01, ****p* < 0.001 compared to cells incabated under control condition without NEFAs (Dunnett’s Multiple Comparison Test). (DOCX 34 kb)

## Abbreviations

GA: gondoic acid
LDs: lipid droplets
MOI: multiplicity of infection
NEFA: non-esterified fatty acid
NTC: non-target control
OA: oleic acid
PA: palmitic acid
ROS: reactive oxygen species
T2DM: type 2 diabetes mellitus
