# Supplementary material for: The role of lipid droplet formation in the protection of unsaturated fatty acids against palmitic acid induced lipotoxicity to rat insulin-producing cells
Source: Nutr Metab (Lond). 2016 Feb 25;13:16. doi: 10.1186/s12986-016-0076-z (PMC4766664; doi:10.1186/s12986-016-0076-z)
Supplement: Additional file 1: Figure S1. — Viability of long-chain unsaturated NEFAs in comparison to the long-chain saturated NEFA PA (C16:0). RINm5F cells were incubated with 100 μM of the long-chain saturated NEFA or 100 μM of the unsaturated NEFAs as depicted for 24 h. Thereafter viability was measured by MTT assay. Data are means ± SEM of n = 5 to 8. *p < 0.05, **p < 0.01, ***p < 0.001 compared to cells incabated under control condition without NEFAs (Dunnett’s Multiple Comparison Test). (DOCX 34 kb) [file 12986_2016_76_MOESM1_ESM.docx]

Additional File 1: Figure S1. Viability of long-chain unsaturated NEFAs in comparison to the long-chain saturated NEFA PA (C16:0)

RINm5F cells were incubated with 100 µM of the long-chain saturated NEFA or 100 µM of the unsaturated NEFAs as depicted for 24 h. Thereafter viability was measured by MTT assay. Data are means ± SEM of n=5 to 8. *p<0.05, **p<0.01, ***p<0.001 (Dunnett´s Multiple Comparison Test).
